# Comparison of Early Outcomes in Patients Undergoing Suture Fixation Versus Tack Fixation of Mesh in Laparoscopic Transabdominal Preperitoneal (TAPP) Repair of Inguinal Hernia

**DOI:** 10.7759/cureus.26821

**Published:** 2022-07-13

**Authors:** Sarmad S Aziz, ZakaUllah Jan, Nadeem Ijaz, Mohammad Zarin, Hamza K Toru

**Affiliations:** 1 Department of General Surgery, Khyber Teaching Hospital, Peshawar, PAK

**Keywords:** suture fixation, tack fixation, mean pain, mesh fixation, inguinal hernia, transabdominal preperitoneal repair

## Abstract

Introduction: The advent of laparoscopic techniques in repairing inguinal hernia has significantly improved outcomes of inguinal hernia surgery. However, acute and chronic postoperative pain after fixation of mesh with tacks and the cost of tacking devices are major hindrances to the widespread use of laparoscopic transabdominal preperitoneal (TAPP) repair in resource-poor settings. This study sought to introduce a method of mesh fixation that will reduce the cost of laparoscopic TAPP repair and might help reduce postoperative pain.

Objective: To compare outcomes in the early postoperative period like pain, seroma, hematoma, urinary retention, and neuralgia after fixation with suture versus the tack fixation of mesh in laparoscopic TAPP repair of inguinal hernia.

Subjects and methods: This study was conducted from 1^st ^June 2019 to 31^st^ May 2020. A total of 144 patients between ages 18 and 60 years with an inguinal hernia on any side and having an American Society of Anaesthesiologists (ASA) score of I/II were included in this study. Patients with a recurrent hernia, large scrotal hernia, strangulated and obstructed hernias, ASA III and ASA IV, prostatism, and chronic cough were excluded. Seventy-two patients were in Group A (tack fixation group) while 72 were in Group B (suture fixation group). Separate investigators were assigned to collect pre-operative and post-operative data from both groups, recorded on specially designed proforma.

Results: The age range was 18 to 60 years with a mean age of 46.53 years ±10.01 S.D in Group A and 46.19 ±9.58 S.D in Group B. In Group A 98.6% of patients were male, and 1.4% were females while in Group B 100% of patients were male. It was found that mean pain in Group A was 4.88 ±0.887 and 5.29± 0.777 at 6 hours and 24 hours respectively. Mean pain in group B was 3.43 ±0.962 and 4.11±0.703 at 6 hours and 24 hours respectively. Moreover, mean pain in Group B was significantly less than mean pain in Group A both at 6 hours and 24 hours intervals with a p-value < 0.001. The early postoperative complications were not significantly different in both groups.

Conclusion: In TAPP repair, suture fixation of mesh is less painful than tack fixation. However, there is no significant difference in the rate of other early postoperative outcomes like seroma, hematoma, urinary retention, and neuralgia. Further multicentric studies with a longer duration of follow-up are needed to validate our results.

## Introduction

Historically, different techniques have been used in the repair of inguinal hernia. Among the many techniques introduced to decrease recurrence and complications associated with hernia repair, laparoscopic techniques have become widely available all over the world. Since the beginning of laparoscopic hernia repair in the early 1990s, many advancements have taken place leading to widespread acceptability of laparoscopic hernia repairs. As a result of the improvements in surgical techniques, prosthetic materials, and a better understanding of their use, the surgical outcome has improved significantly. The problems associated with hernia surgery are postoperative pain, prolonged hospital stay & recurrence. Compared to open surgery the laparoscopic technique is associated with a short period of stay and early return to work [1]. Thus in the last 15 years, laparoscopic hernia repair has become a real option with recurrence rates of less than 1% and minimal long-term discomfort [2].

TAPP (transabdominal preperitoneal) repair and TEP (totally extraperitoneal) repair are the two most commonly performed procedures for the repair of laparoscopic hernia. In TAPP repair, a peritoneal flap is raised after gaining entry into the abdominal cavity, and the preperitoneal space is used for placing the mesh. Different materials are used for the fixation of mesh against the anterior abdominal wall including transfascial sutures (absorbable and non-absorbable), titanium tacks, fibrin glue, and synthetic sealants, all of which have advantages and disadvantages [3-5]. Titanium tacks are usually used for fixation of the mesh to the anterior abdominal wall, but they are also associated with complications such as nerve entrapment, erosion into the bowel and other hollow viscera as well as the formation of dense adhesions and so-called tack hernias [6]. However acute and chronic postoperative pain have emerged as clinically most relevant negative impact factors. Moreover, titanium tacks are costly, making laparoscopic inguinal hernia repair unaffordable for our poor patients. To address the problem of postoperative pain and cost-effectiveness non-fixation of Mesh has been attempted; however, with non-fixation, the probability of mesh migration increases as reported in several cases in the literature [7]. Another novel method involves the use of vicryl 2/0 for mesh fixation which is cost-effective and has decreased probability of mesh migration and incidence of recurrence at six months compared to tack fixation [4].

Since early postoperative pain has become an important factor in the evaluation of different laparoscopic techniques, this study intends to compare early postoperative pain in tack fixation versus vicryl fixation in addition to seroma, hematoma, urinary retention, and neuralgia.

During the literature review, two studies were found comparing early postoperative pain in suture fixation versus tack fixation of mesh in TAPP repair, these studies show lesser postoperative pain and analgesic consumption with suture fixation compared to tack fixation [4-5]. The mean pain score on day 1 in the suture group (4.63±1.59) was found significantly lower than the tacker group (5.54±1.68) with a p-value of 0.0244. A similar study conducted by Oguz et al. compared suture versus tack as a peritoneum closure technique and also showed lesser postoperative pain with suture closure (1.7±0.2) compared to tack closure (2.4±0.2) with a p-value of 0.027 [8]. However, studies comparing fixation of the mesh with suture versus tack in hernias of the ventral wall show no significant difference in pain after the operation following suture versus tack fixation p=0.38 [9-10].

This study would primarily compare the early postoperative pain after suture fixation versus fixation of mesh with tack in TAPP repair in addition to other early postoperative outcomes. As there are not enough studies on this topic both in international as well as local journals, our study is intended to clarify the controversy of whether suture fixation of mesh in TAPP repair causes lesser postoperative pain or vice versa. 

## Materials and methods

This study was conducted in the Surgical 'C' unit, Khyber Teaching Hospital, Peshawar, from 01/6/2019 to 31/05/2020. The total sample size was 144 (72 in each group) with an allocation ratio of 1:1. The sampling technique was consecutive non-probability sampling. Institution Research and Ethical Review Board (IREB) Khyber Medical College Peshawar approved this study with an approval number 214/ADR/KMC.

All patients between 18 and 60 years with unilateral inguinal hernia, diagnosed on physical examination, presenting to the Surgical 'C' unit at Khyber Teaching Hospital who opt to undergo laparoscopic hernia repair and had no comorbidity that contraindicates general anesthesia and laparoscopic surgery were included in this study. Patients with a recurrent hernia, large scrotal hernia, strangulated and obstructed hernias, American Society of Anaesthesiologists (ASA) III and ASA IV, prostatism, and chronic cough were excluded.

After approval from the independent ethics committee of the Khyber Teaching Hospital, all patients meeting the inclusion criteria were admitted from OPD in the Surgical ‘C’ unit, Khyber Teaching Hospital Peshawar. A clinical diagnosis of hernia was made on physical examination. All patients with a reducible swelling in the inguinal region and positive cough impulse were diagnosed as having inguinal hernia that was confirmed on ultrasonography. The purpose of the study was explained to all patients, and written informed consent was obtained from them.

**Figure 1:**
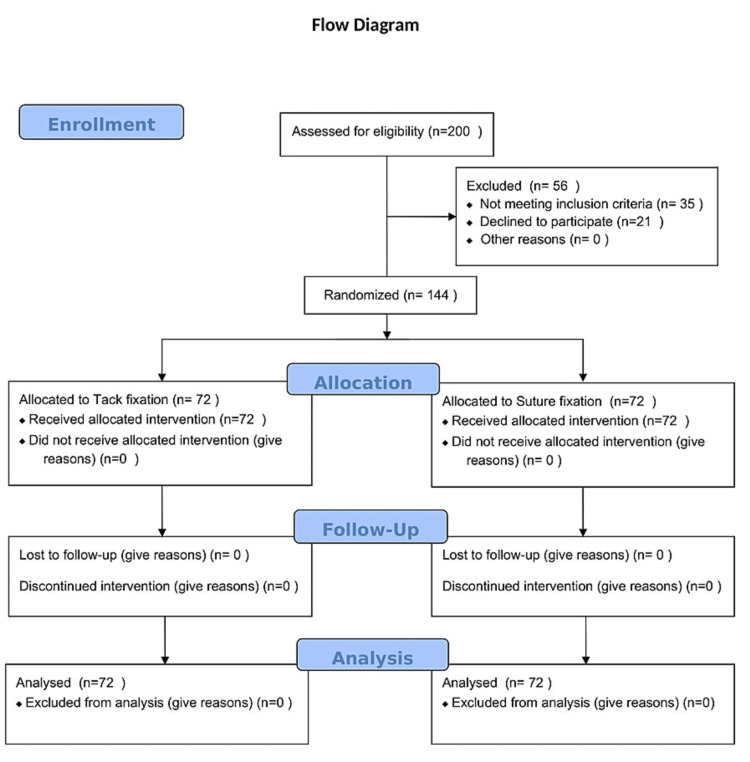
Consort 2010 Flow Diagram

A detailed history was taken from all patients followed by their clinical examination and routine investigations. All the patients were randomized into two groups by the block randomization method. Patients undergoing tack fixation of mesh were placed in group A and those undergoing suture fixations were placed in group B (Figure 1).

An investigator was assigned to collect preoperative data. All the preoperative information including age, gender, weight, years of education, and the group assigned was recorded on a predesigned proforma. A surgeon trained in advanced laparoscopy and an expert in laparoscopic TAPP repair performed the procedure.

Surgery was performed using general anesthesia. Transabdominal Preperitoneal Mesh Hernioplasty (TAPP) was carried out in all patients. In this technique, three laparoscopy ports are used that give access to intraperitoneal space from where preperitoneal space is approached by raising peritoneal flaps, and dissection is done in the inguinal region to separate the hernial sac from its contents. Then mesh is applied and is fixed to the anterior abdominal wall using different techniques such as titanium tacks, fibrin glue, and sutures, and the space is closed again by stitching together the peritoneal flaps. In our study, the suture fixation group underwent mesh fixation by taking three stitches with vicryl 2/0 against the anterior abdominal wall and coopers ligament for anchoring the mesh. Taking stitches in the triangle of doom and the triangle of pain were specifically avoided. The tack fixation group underwent mesh fixation with three titanium tacks taken against the anterior abdominal wall and coopers ligament. Taking tacks in the triangle of doom and triangle of pain was specifically avoided. Post-operatively all patients were administered the same analgesics; injection ketorolac 30mg three times a day and tablet diclofenac sodium 50mg as required.

Post-operative pain was compared at 6 hours postoperatively and 24 hours postoperatively by recording the mean pain score on the visual analogue scale (VAS). Data were analyzed using SPSS version 23.0 (IBM Corp., Armonk, NY). Mean ± SD was calculated for quantitative variables like age, visual analogue score, years of education, and body weight. Frequencies and percentages were calculated for categorical variables like gender in both groups (suture fixation and tack fixation). Independent samples t-test was used to compare VAS among both groups at two different intervals (6 hrs and 24 hrs). The mean pain score of both groups was stratified by age, gender, years of education, and weight to see effect modifiers using an independent sample t-test. A p-value ≤ 0.05 was considered significant.

## Results

The study had 144 participants who presented with inguinal hernia ranging in age from 18 to 60 years (mean age of 46.36 years). The study groups were found statistically similar in terms of demographics (Table 1).

**Table 1:** Patient demographics

	Group A (Tack fixation)	Group B (Suture fixation)	P-Value
Male	71	72	0.316
Female	1	0	
Age(mean±SD)	46.53±10.01 yrs	46.19±9.58 yrs	0.839
Weight (mean±SD)	73.49±6.88 kg	74.51±6.74 kg	0.367
Years of Education (mean±SD)	10.4±4,88 yrs	9.03±4.98 yrs	0.097

Mean pain distribution among two groups at 6 hours and 24 hours postoperatively was analyzed. The mean pain score at 6 hours in Group B (3.43± 0.962) was significantly less than the mean pain in Group A (4.88 ± 0.887). The mean postoperative pain score at 24 hours in group B (4.11± 0.703) was also significantly less than that in Group A (5.29 ± 0.777) (Table 2).

**Table 2:** Mean pain at 6 and 24 hours (n=144)

Mean Pain	Group A (Tack fixation) (n=72)	Group B (Suture fixation) (n=72)	P-value
At 6 hours	4.88 ± 0.887	3.43± 0.962	<0.001
At 24 hours	5.29 ± 0.777	4.11± 0.703	<0.001

The early postoperative complications are given in Table 3. There was no significant difference in other early postoperative outcomes between the groups. The postoperative complications occurred in seven patients in Group A and 2 patients in Group B.

**Table 3:** Early postoperative complications

Complication	Group A	Group B	
Hematoma formation	0	0	
Urinary retention	3	1	
Seroma formation	2	0	
Scrotal edema	2	1	
Neuralgia	0		0

## Discussion

Advancements in laparoscopic surgical techniques have led to widespread acceptance of laparoscopic inguinal hernia repair among surgeons and patients. Patients who underwent laparoscopic inguinal hernia repair have less postoperative pain and tend to return early to work [11-15].

Transabdominal preperitoneal (TAPP) repair and totally extraperitoneal (TEP) repair are the two commonly used laparoscopic techniques of inguinal hernia repair. After dissecting the hernia sac, both techniques use the preperitoneal space for mesh placement. Thereafter, the mesh is secured against the anterior wall of the abdomen. The crucial step in an inguinal hernia repair is the fixation of mesh which is associated with vascular and neurological complications [16,17]. Most commonly, the mesh is tethered against the abdominal wall with titanium tacks. Titanium tacks have been associated with complications such as nerve entrapment, erosion into the bowel and other hollow viscera, and formation of dense adhesions and so-called tack hernias [6,7]. In previous studies, chronic neuralgia has been reported in approximately 14 % of hernia repairs that used titanium tacks [18-20]. The most vulnerable nerves subject to injury during laparoscopic repair are the lateral cutaneous nerve of the thigh, genitofemoral nerve, the iliohypogastric, and the ilioinguinal nerves [1]. In the mentioned nerves, the nerve known to be injured in particular is the lateral cutaneous nerve (0.1 -10 % of cases) [21-24]. The nerve injury occurs due to entrapment of the nerve in the staple tacks. Stark et al. reported a 4.2% incidence of neuralgia [25]. Suprapubic or pelvic pain has been reported in some trials following TAPP repair [1]. Previously, two cases have been reported having neural complications among 1514 patients undergoing TAPP repair [26]. Moreover, the prevalence of neuralgia was reported at 2% and the incidence of chronic pain was 0.4% among 9955 undergoing TAPP repairs [27].

Based on the findings in the above citations, it is evident that postoperative pain and neuralgia can be expected after TAPP repair. In order to tackle the problem of postoperative pain and neuralgia after fixation of mesh with titanium tacks, different methods are being used such as changing the orientation of endotacker to vertical while placing tacks and applying a smaller number of tacks [16,17,28]. Ferzli et al. showed that fixation of the mesh to the abdominal wall is not required [29]. However, with the non-fixation, the probability of mesh migration increases in several cases reported in the literature [7]. Different authors have reported alternative mesh fixation methods such as absorbable tacks, human fibrin glue (tissucol), synthetic sealants, and transfascial absorbable and non-absorbable sutures [1,3-5]. These methods are reported to be associated with less postoperative pain and neuralgia [4,5,30].

In our study, we compared pain following suture fixation of the mesh versus tacker fixation. In our study, the early postoperative pain was found to be significantly less in suture fixation as compared to tack fixation (p<0.001). This proved our hypothesis that the mean pain after suture fixation and tack fixation are not equal. Another study by Kleidari et al. showed that the in-hospital mean pain score recorded on the morning after surgery was not significantly different between suture fixation and tacker fixation. However, Kleidari et al. found that mean pain at day one and day six after discharge was significantly less in the suture group than in the tacker group which supports our findings [4]. Another study from Egypt reported no significant postoperative pain in patients who underwent TAPP repair involving fixation of mesh with transfascial sutures [5].

Oguz et al. reported that the statistical comparison of the patients in whom tacker was used for fixation did not affect VAS 1 (VAS on the first postoperative day). Conversely, VAS 1 was significantly lower in patients where sutures were used for peritoneal closure than in patients where tacker was used for peritoneal closure (p = 0.027) [8]. The finding of Oguz et al. was that mesh fixation with 1 or 2 tacks does not significantly affect postoperative pain on day one which contradicts our results. This may be due to the lesser number of tacks used for mesh fixation by Oguz et al. However, the findings of Oguz et al. that postoperative pain on day one is significantly less in suture closure of peritoneum as compared to tacker closure of peritoneum indirectly supports our finding that use of tacker is associated with more pain compared to suture.

On the other hand, studies comparing fixation of the mesh with suture against tack fixation in ventral hernias showed no difference in postoperative pain between the suture and tack fixation p=0.38 [9,10].

Strengths and limitations

The strength of our study is that no significant difference existed between the two groups with respect to age, sex, weight, educational status, and the number of tackers used for mesh fixation. Also, our study was double-blind with neither the patient knowing which technique they underwent nor the investigators as different investigators were assigned to collect preoperative and postoperative data. Also, a single surgeon was assigned to carry out all the operations, so that the operative technique and surgical skill of the surgeon did not act as an effect modifier.

The limitation of our study is that it is a single-centered study. Also, the sampling technique used was non-probability consecutive sampling. Moreover, while comparing the mesh fixation methods, only early outcome variables were studied.

## Conclusions

In laparoscopic transabdominal preperitoneal repair, suture fixation of the mesh is less painful than tack fixation of the mesh. However, there is no statistically significant difference in the rate of other early postoperative complications. Further multicentric studies with a longer duration of follow-up are needed to validate our results. In addition, other important outcome variables such as chronic pain, hernia recurrence, and incidence of mesh migration need to be compared in both techniques.
